# Autophagy Is a Potential Therapeutic Target Against Duck Tembusu Virus Infection *in vivo*

**DOI:** 10.3389/fcimb.2020.00155

**Published:** 2020-04-15

**Authors:** Zhiqiang Hu, Yuhong Pan, Anchun Cheng, Xingcui Zhang, Mingshu Wang, Shun Chen, Dekang Zhu, Mafeng Liu, Qiao Yang, Ying Wu, Xinxin Zhao, Juan Huang, Shaqiu Zhang, Sai Mao, Xumin Ou, Yanling Yu, Ling Zhang, Yunya Liu, Bin Tian, Leichang Pan, Mujeeb Ur Rehman, Zhongqiong Yin, Renyong Jia

**Affiliations:** ^1^Institute of Preventive Veterinary Medicine, Sichuan Agricultural University, Wenjiang, China; ^2^Avian Disease Research Center, College of Veterinary Medicine of Sichuan Agricultural University, Wenjiang, China; ^3^Key Laboratory of Animal Disease and Human Health of Sichuan Province, Sichuan Agricultural University, Wenjiang, China

**Keywords:** DTMUV, autophagy, spleen, brain, tissue damage, replication, immune response

## Abstract

Duck tembusu virus (DTMUV) is newly emerged in poultry and causes great losses to the breeding industry in China and neighboring countries. Effective antiviral strategies are still being studied. Autophagy is a cellular degradative pathway, and our lab's previous data show that autophagy promotes DTMUV replication *in vitro*. To study the role of autophagy further *in vivo*, we utilized ducks as the animal model to investigate the autophagy responses in DTMUV-targeted tissues. And also, we utilized autophagy regulators, including Rapamycin (Rapa) as the autophagy enhancer, 3-Methyladenine (3-MA) and Chloroquine (CQ) as the autophagy inhibitors, to adjust the host autophagic levels and then study the effects of autophagy on tissue damages and virus replication. As a result, we first found DTMUV infection trigged autophagy and autophagy regulator treatments regulated autophagy levels successfully in duck spleens and brains. Next, we found that autophagy inhibitors inhibited DTMUV replication and alleviated DTMUV-induced pathological symptoms, whereas the autophagy inducer treatment led to the opposite effects. And we also found that autophagic regulation was correlated with the expression of innate immune genes, including pattern recognition receptors, type I interferons, and cytokines, and caused different effects in different tissues. In summary, we demonstrated that autophagy facilitated DTMUV replication, aggravated the developments of pathological symptoms and possibly counteracts the host's innate immunity response *in vivo*.

## Introduction

Autophagy is a cellular degradative pathway that delivers intracellular senescent organelles, long-lived proteins, and exogenous pathogenic microorganisms to lysosomes for degradation (Klionsky and Emr, 2000). When cells are exposed to environmental stresses such as starvation, lack of growth factors, energy requirements, or invasion by pathogenic microorganisms, autophagy will be activated as a defense strategy to maintain the homeostasis of the intracellular environment (Chun and Kim, 2018). Autophagy is a dynamic process, including the formation of autophagosomes and the degradation of autophagosomes in lysosomes. One of the key signals for the formation of autophagosome is the mammalian target of rapamycin complex 1 (mTORC1), which is suppressive for autophagy (Klionsky et al., 2016). Therefore, some mTOR inhibitors have been used as autophagy inducers, such as Rapa (Jung et al., 2010) and Everolimus (Crazzolara et al., 2009). Another critical signal is phosphatidylinositol 3-kinases (PI3K), which is required for autophagy. And some PI3K inhibitors have been used as autophagy inhibitors, such as 3-MA (Wu et al., 2010), wortmannin (Blommaart et al., 1997), and 2-(4- morpholinyl)-8-phenyl-4H-1-benzopyran-4 -one (LY294002) (Blommaart et al., 1997). There are also some autophagy inhibitors targeting the process of autophagic degradation. For instance, Chloroquine (CQ) has been reported to inhibit autophagy by raising the lysosomal pH, which leads to inhibition of both fusion of autophagosome with lysosome and lysosomal protein degradation (Shintani and Klionsky, 2004). Bafilomycin A1 also inhibits autophagic degradation by inhibiting fusion between autophagosomes and lysosomes (Yamamoto et al., 1998).

Duck Tembusu virus (DTMUV), a member of the *Flavivirus* genus within the Flaviviridae family, is newly emerged in ducks in China (Su et al., 2011; Yan et al., 2011). Duck is the typical host of DTMUV, and some other species, like chickens, geese, and sparrows, also have been reported to be infected with DTMUV (Yu et al., 2018). Seriously, there might be high risk for poultry-to-human or mice transmission of the DTMUV (Liu et al., 2013; Tang et al., 2013). Therefore, it is emergency to study the DTMUV-host interaction and develop effective anti-virus therapies. Multiple evidence has shown that the duck spleen is the target organ of DTMUV (Li et al., 2015; Sun et al., 2019b). Moreover, DTMUV has been reported to cause neurologic dysfunction (Thontiravong et al., 2015; Lv et al., 2019), which is similar to the neurological symptom caused by other flavivirus (Mustafá et al., 2019). And the presence of DTMUV has been detected in the duck brain (Li et al., 2015; Lv et al., 2019), which indicates that the duck brain is another target organ of DTMUV.

Multiple evidence has indicated that autophagy plays an important role in flavivirus infection (Ke, 2018). But there are rare reports on the effect of autophagy on virus replication *in vivo*. In this study, we first utilized ducks as the animal model to invested the role of autophagy in DTMUV infection *in vivo*.

## Materials and Methods

### Ethics Statement

All animal experiments have been approved by the committee of experiment operational guidelines and animal welfare of Sichuan Agricultural University, China (the approved permit number is XF2014-18) and were performed in accordance with the relevant guidelines and regulations of National Institutes of Health. All surgeries were performed under sodium anesthesia with pentobarbital and every effort was made to relieve the suffering.

### Animals and Virus

One-day-old Cherry Valley ducks were purchased from a farm operated by Sichuan Agricultural University (Sichuan, China) and housed in isolators until use. DTMUV-free ducks were confirmed by an immunochromatographic strip (ICS) developed by our lab (Deng et al., 2017). The DTMUV CQW1 strain (GenBank: KM233707.1) used in this study was isolated from a young duck in Southwest China and purified by the plaque method in our laboratory (Zhu et al., 2015).

### Experimental Design

Twenty-five 7-day-old ducks were randomly divided into 5 groups (*n* = 5/each group). The ducks in group 2, 3, 4, and 5 were infected with 400,000 TCID50 viruses by intramuscular injection, and then treated with saline, rapamycin (Rapa, 2 mg/kg of body weight), 3-Methyladenine (3-MA, 2 mg/kg), or Chloroquine (CQ, 20 mg/kg) by intraperitoneal injection, respectively. The pharmaceutical treatments were carried out 2 h after virus infection, which was followed by treatments with drugs or saline every 12 h. The ducks in group 1 were treated with saline as the control. At 72 h posttreatment, these ducks were euthanized and duck tissues were collected for different goals with different protocols as followed.

### Antibodies and Chemicals

The primary antibodies of anti-LC3 (14600-1-AP) and anti-β-actin (60008-1-Ig), were purchased from Proteintech (Wuhan, Hubei, China). Anti-SQSTM1/p62 (5114) was purchased from Cell Signaling Technology (Danvers, Massachusetts, USA). The monoclonal antibody against the DTMUV E protein was prepared in our laboratory. Horseradish peroxidases (HRP) conjugated to goat anti-mouse secondary antibodies (BF03001) were purchased from Beijing Biodragon Immunotechnologies (Beijing, China). Rapamycin (Rapa) (HY-10219), 3-Methyladenine (3-MA) (HY-19312), chloroquine (CQ) (HY-17589), and were purchased from MedChemExpress (MCE, Monmouth Junction, New jersey, USA).

### Western Blotting (WB)

Hundred milligram of spleens specimens and brains specimens were weighed and then immediately cryopreserved in liquid nitrogen until being processed for protein isolation. When processed for protein isolation, spleen tissues and brain tissues were homogenized and then lysed with RIPA lysis buffer (Solarbio, R0020, Beijing, China) containing 1 mM phenylmethylsulfonyl fluoride (PMSF, an inhibitor of serine proteases and acetylcholinesterase) (Boster, AR1178, Beijing, China). The concentration of extractive protein was measured using a BCA protein assay kit (Solarbio, PC0020, Beijing, China). Equal amounts of protein samples were boiled for 5 min in 4 × SDS-PAGE loading buffer, separated on 12-15% SDS-PAGE gels, and then electrotransferred onto polyvinylidene fluoride (PVDF) membranes (BIO-RAD, 162-0177, Hercules, California, USA). The PVDF membranes with the target proteins were then blocked for 2 h at room temperature in Tris-Buffered Saline and Tween 20 (TBST) containing 5% non-fat milk powder. After that, the membranes were incubated with anti-LC3 (1:1000), anti-p62 (1:1000) and anti-β-actin (1:2000) antibodies at 4 °C overnight and then with the corresponding secondary antibodies (1:5000), conjugated to HRP at 37 °C for 1 h. The protein bands were developed by an ECL Plus kit (Solarbio, PE0010, Beijing, China) and imaged by ChemiDoc MP (Bio-Rad, Hercules, California, USA). The densitometry of WB bands was measured by the Image Lab software.

### Hematoxylin and Eosin (HE) Staining and Immunohistochemistry (IHC)

The spleen tissues and brain tissues were fixed in 4% paraformaldehyde, and then enclosed in paraffin-intended subsequent histopathological examination. A 4 μm section of each tissue was stained with hematoxylin and eosin. Each section was examined under an optical microscope. IHC was conducted as described previously (Ou et al., 2017). Briefly, slides were boiled in Tris/EDTA pH 9.0 for 20 min. Then, 0.01 M HCl was used to block endogenous alkaline phosphatase for 15 min at room temperature (RT). Then, 3% H_2_O_2_ was used to block endogenous peroxidase for 15 min at RT. The slides were incubated in 5% BSA blocking solution followed by overnight incubation at 4°C in mouse anti-DTMUV-E polyclonal antibody (1:20 dilutions). HRP coupled goat anti-mouse IgG (1:1000 dilutions) was incubated for 30 min at 37°C. Then, positive staining was colored with DAB solution for 10 min at RT and counterstained with hematoxylin.

### Quantitative RT-PCR Assay (qRT-PCR)

Hundred milligram of spleen specimens and brains specimens were collected with the same procedure as previously described in the section of “**Western blotting**.” Total cellular RNA was isolated from 100 mg of tissue specimens using the RNAiso plus Reagent (TaKaRa, Japan), and subsequently transcripted into cDNA using PrimeScript™ RT reagent Kit (Takara, RR047A, Dalian, China) according to the manufacturer's protocol. The mRNA levels of immune genes, including RIG-I, MDA5, TLR3, IFN-α, IFN-β, IFN-γ, IL-1β, IL-6, and IL-8, were detected by qRT-PCR which was performed using the Bio-Rad CFX96 Real-Time Detection System (Bio-Rad, USA), and the β-actin was as the housekeeping gene. Additionally, viral copies were detected by previously established methods in our laboratory (Zhang et al., 2019b). The sequences of the gene-specific primers used for qRT-PCR are shown in Table 1.

**Table 1:** Primer sequences used in this study.

**Prime name**	**Prime sequence(5^**′**^-3^**′**^)**
Duck Rig-I-F	GCGTACCGCTATAACCCACA
Duck Rig-I-R	CCTTGCTGGTTTTGAACGC
Duck MDA5-F	GCTGAAGAAGGCCTGGACAT
Duck MDA5-R	TCCTCTGGACACGCTGAATG
Duck TLR3-F	GAGTTTCACACAGGATGTTTAC
Duck TLR3-R	GTGAGATTTGTTCCTTGCAG
Duck IFN-α-F	TCCTCCAACACCTCTTCGAC
Duck IFN-α-R	GGGCTGTAGGTGTGGTTCTG
Duck IFN-β-F	AGATGGCTCCCAGCTCTACA
Duck IFN-β-R	AGTGGTTGAGCTGGTTGAGG
Duck IFN-γ- F	GCTGATGGCAATCCTGTTTT
Duck IFN-γ R	GGATTTTCAAGCCAGTCAGC
Duck IL-1β-F	TCATCTTCTACCGCCTGGAC
Duck IL-1β-R	GTAGGTGGCGATGTTGACCT
Duck IL-6-F	TTCGACGAGGAGAAATGCTT
Duck IL-6-R	CCTTATCGTCGTTGCCAGAT
Duck IL-8-F	AAGTTCATCCACCCTAAATC
Duck IL-8-R	GCATCAGAATTGAGCTGAGC
Duck β-actin-F	GGTATCGGCAGCAGTCTTA
Duck β-actin R	TTCACAGAGGCGAGTAACTT
DTMUV-Cap-F	AGGTTTGTGCTGGCTCTAC
DTMUV-Cap-R	TGTTTGGTCGCCTCATT

### Statistical Analysis

Data are presented as means ± standard deviations (SD). The significance of the variability between different treatment groups was analyzed by the two-tailed independent Student *t*-test using the GraphPad Prism software (version 6.0). A *P* < 0.05 was considered to be statistically significant.

## Results

### DTMUV Infection Enhances Autophagy in Duck Spleens and Brains

**Figure 1:**
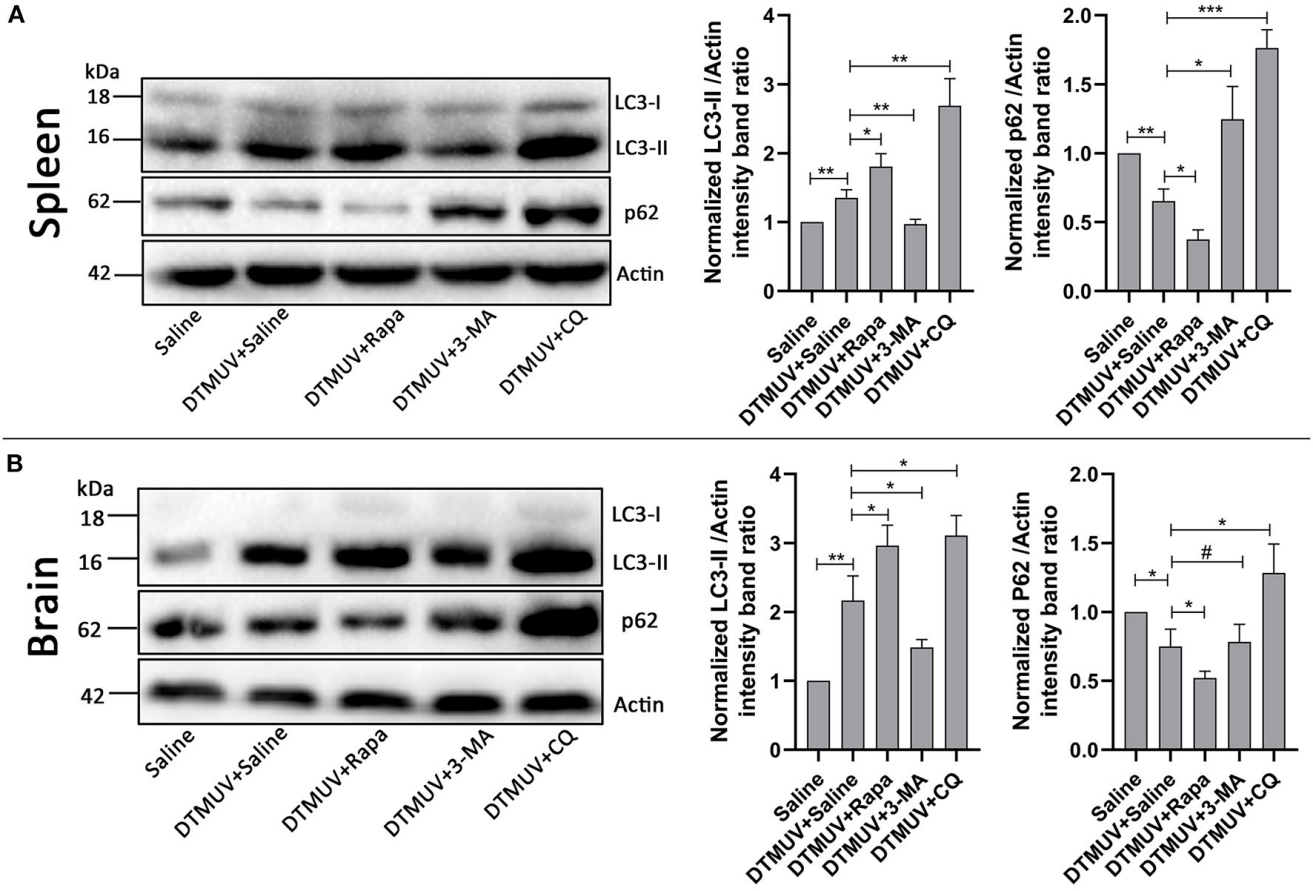
Measurement of autophagic markers in the duck spleens and brains. The protein levels of LC3 and p62 in duck spleens **(A)** and duck brains **(B)** were analyzed by Western blot assay. The ratios of targeting proteins to β-actin were normalized to those in the saline group. Data are expressed as means ± standard deviations (*n* = 5). Differences were evaluated with Two-tailed Student's *t*-test. ^#^*p* > 0.05, *p < 0.05, **p < 0.01, ***p < 0.001.

Our previous data have shown that DTMUV infection triggered autophagy *in vitro*. To further know the role of autophagy *in vivo*, we tested the protein levels of autophagy makers, including the conversion from LC3-I to LC3-II and the degradation of p62, in DTMUV-targeted organs. Duck spleens and brains have been reported to be the target organs in DTMUV infection (Li et al., 2015; Thontiravong et al., 2015; Lv et al., 2019; Sun et al., 2019b). As shown in Figure 1, DTMUV infection increased the protein levels of LC3-II but decreased p62 levels compared to those in the control group, which indicated that DTMUV enhanced both autophagic initiation and autophagic flux in spleens (Figure 1A) and brains (Figure 1B). We also examined the formations of autophagosome-like vesicles in the two organs by TEM. Many double-membrane structures, including autophagosome-like (Figures S1C,G) and autolysosome-like vesicles (Figures S1D,H), were observed in the spleens and brains of DTMUV-infected ducks (Figures S1B,F), whereas few double-membrane structures observed in the organs of saline-treated ducks (Figures S1A,E). Furthermore, we found that Rapa treatment further improved LC3-II increase and p62 decrease in both spleens and brains (Figures 1A,B), which indicated the promotion of autophagic activation. 3-MA treatment, an inhibitor of autophagy initiation, decreased the levels of LC3-II but increased p62 levels in spleens compared to those in DTMUV-infected ducks with saline treatment. Whereas, there were decreased levels of LC3-II, but no significant change of p62 levels in brains (Figure 1B). CQ treatment enhanced the accumulation of both LC3-II and p62, which indicated that DTMUV-trigged autophagic flux was inhibited in both spleens and brains.

### Autophagy Regulators Affect the Gross Pathology of Spleens and Brains in DTMUV-Infected Ducks

**Figure 2:**
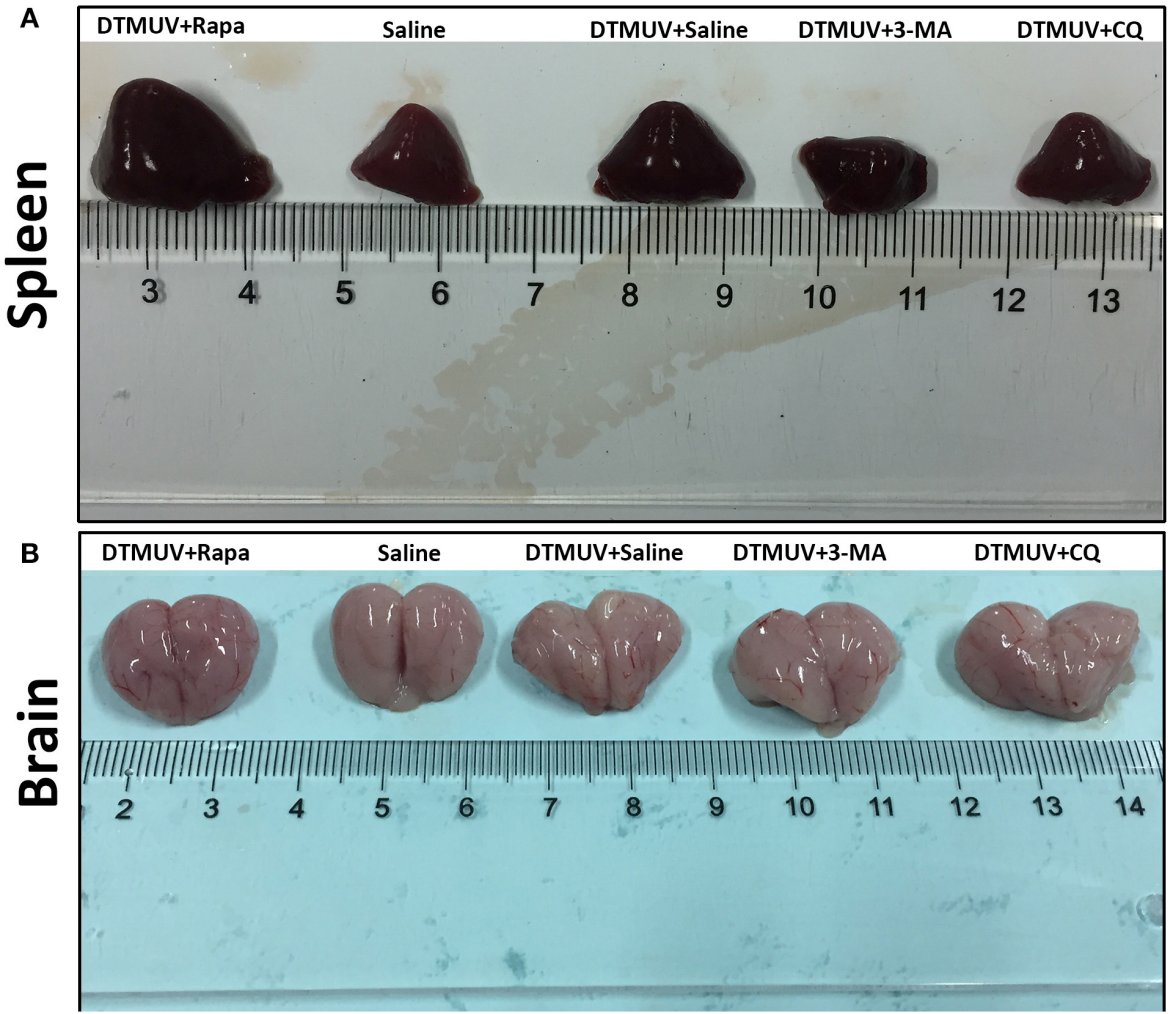
Gross pathology of the spleens **(A)** and the brains **(B)** of ducks infected with DTMUV in the absence or presence of either Rapa, 3-MA, or CQ, respectively. Treatment with saline was used as the control. Images shown were representative from five ducks in each group.

To investigate the effect of autophagy on the gross pathology of spleens and brains in DTMUV-infected ducks, we observed the gross pathologies of the two tissues with different autophagy regulator treatments. As shown in Figure 2A, the normal spleen was with normal size and in pale red. DTMUV infection caused the spleen to be swollen and in dark red. Rapa treatment aggravated these symptoms, whereas 3-MA and CQ treatments relieved them. As for brains, as shown in Figure 2B, the normal brain was in light pink and with few blood streaks on the meninx. DTMUV infection caused meninx congestion, and Rapa treatment made the brain dark pink, which indicated more serious symptoms in the brain. Whereas, there were fewer blood streaks on the brains with 3-MA and CQ treatments, which indicated that the symptom of meninx congestion was relieved.

### Autophagy Regulators Affect the Histopathologic Lesions of Spleens and Brains in DTMUV-Infected Ducks

**Figure 3:**
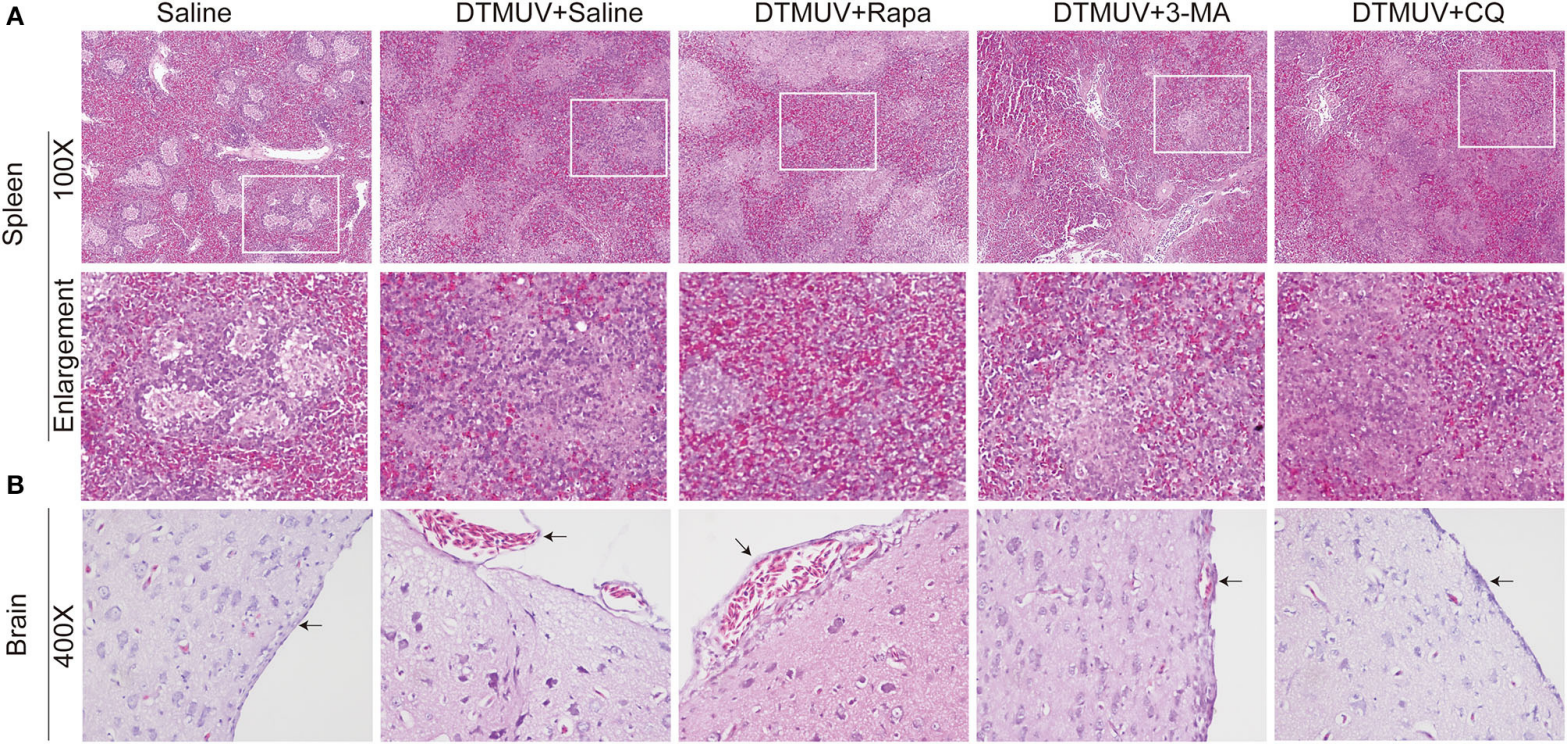
Hematoxylin and eosin staining of cells in the spleens **(A)** and the brains **(B)** of ducks infected with DTMUV in the absence or presence of either Rapa, 3-MA, or CQ, respectively. Treatment with saline was used as the control. Images shown were representative from five ducks in each group. The images were with 100X magnification for spleens and 400X for brains. Black arrows: blood cells in the meninx.

We further analyzed the histopathologic lesions of spleens and brains. As shown in Figure 3A, there were normal structures of the spleens in the only saline-treated ducks, including white pulps (WPs), red pulps (RPs), and the clear boundary line between WPs and RPs. DTMUV infection caused an obvious increase of red blood cells in RPs, which indicated the spleen congestion and hemorrhage. This result might explain the dark red color of spleens with DTMUV infection (Figure 2A). Rapa treatment aggravated these pathological changes, and it was hard to observe the boundary line between WPs and RPs in these spleens. On the contrary, there were just slight congestions in the spleens with 3-MA treatments and CQ treatments, which indicated that 3-MA and CQ alleviated these pathological symptoms caused by DTMUV infection.

As for brains (Figure 3B), there were normal meninx structure, and no blood cells observed under the meninx. While the space under meninx was dilated and amounts of blood cells were observed in that in DTMUV-infected brains. And Rapa treatment further aggravated the histopathologic lesions induced by DTMUV infection. There was a bigger space under meninx and the blood might outflow of the meninx in the DTMUV-infected brains with Rapa treatment. On the contrary, there were just slight swellings of meninges and a few blood cells under the meninges in the DTMUV-infected brains with 3-MA treatments and CQ treatments.

### Autophagy Regulators Affect the Replication of DTMUV in Duck Spleens and Brains

**Figure 4:**
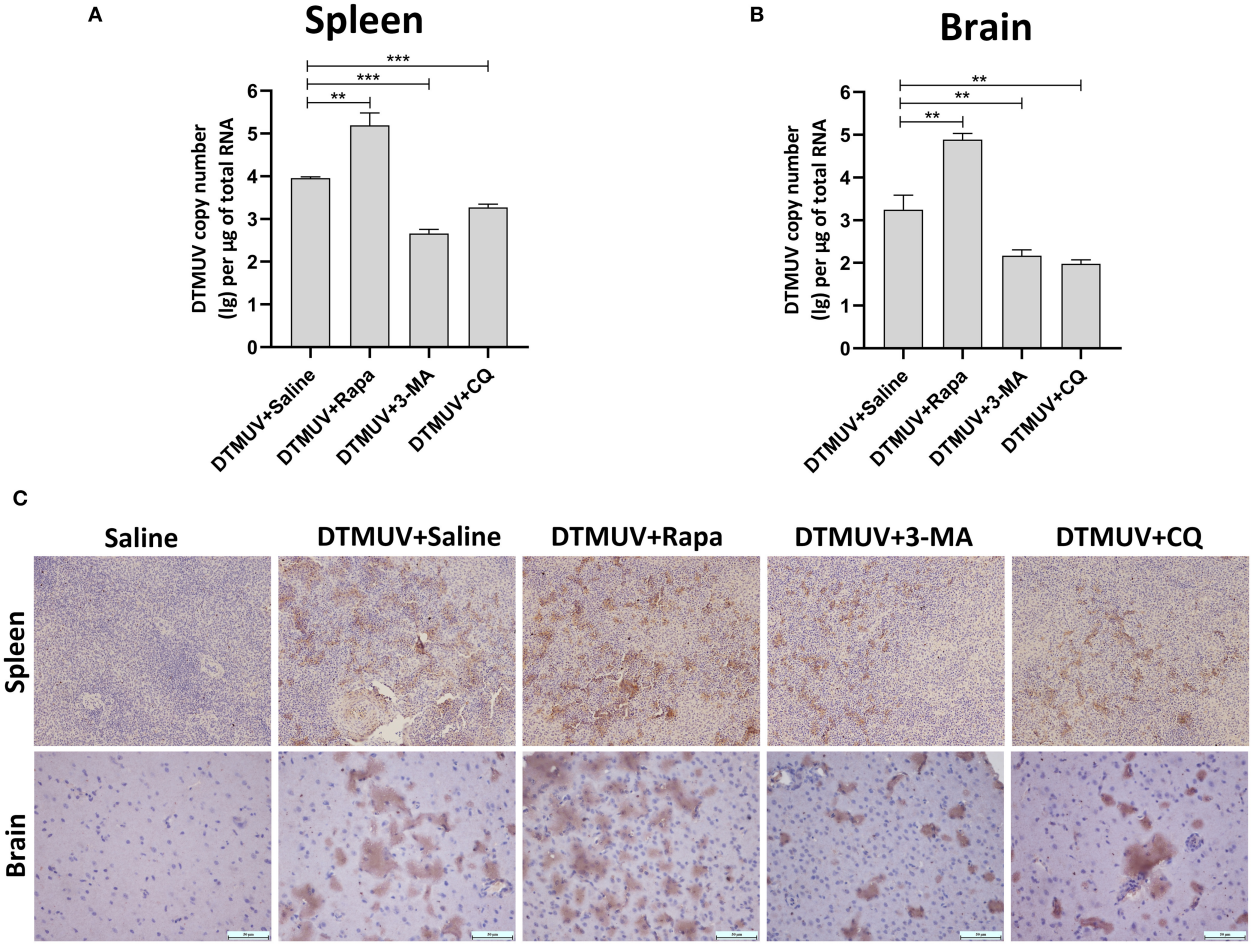
The replication of DTMUV in the spleens and the brains of ducks infected with DTMUV in the absence or presence of either Rapa, 3-MA, or CQ, respectively. **(A,B)** The viral titers in the spleens **(A)** and the brains **(B)** were analyzed by qRT-PCR and expressed as means ± standard deviations (*n* = 5). Differences were evaluated with Two-tailed Student's *t*-test. **p < 0.01, ***p < 0.001. **(C)** The expression of DTMUV E protein was tested by IHC. Images shown were representative from five ducks in each group.

Generally, the levels of pathological symptoms were always related to the amount of virus in tissues. Then, we analyzed DTMUV replication in spleens and brains of ducks with autophagy regulator treatments. First, the virus titers were analyzed by qRT-PCR. As shown in Figures 4A,B, the results showed that virus titers were increased significantly in both spleens and brains with Rapa treatments, whereas decreased with 3-MA and CQ treatments compared to that in the only saline-treated group. Furthermore, the expression of envelope protein of DTMUV was tested by IHC in the two tissues. As shown in Figure 4C, many E-positive cells were observed in DTMUV-infected spleens and brains with Rapa treatment or not. While, there was a decreased number of E-positive cells in 3-MA-treated and CQ-treated tissues. No E-positive cells were observed in only saline-treated spleens or brains.

### Autophagy Regulators Affect the Innate Immune Responses of Spleens and Brains in DTMUV-Infected Ducks

Autophagy has been reported to play essential roles in virus infection and host immune responses. To investigate the effects of autophagy on the innate immune responses with DTMUV infection *in vivo*, we tested the mRNA expression levels of pattern recognition receptors (PRRs, including RIG-1, MDA5, and TLR3), interferons (IFNs, including IFN-α, IFN-β, and IFN-γ) and cytokines (IL-1β, IL-6, IL-8), in duck spleens and brains.

#### Expression of Immune Genes in the Spleens

**Figure 5:**
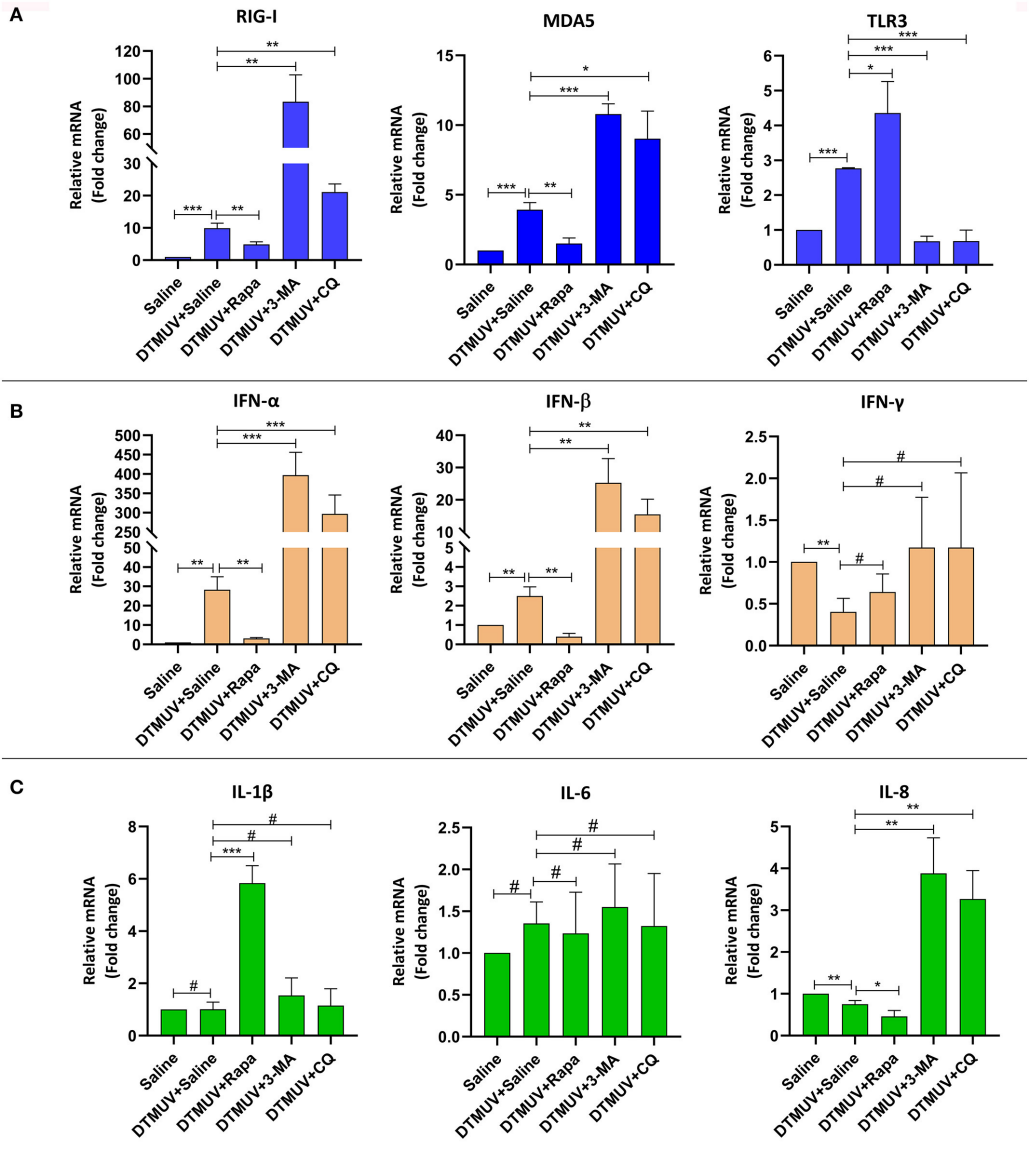
Expression of immune-related genes in the spleens of ducks infected with DTMUV in the absence or presence of either Rapa, 3-MA, or CQ, respectively. **(A)** The mRNA levels of PRRs, including RIG-I, MDA5, and TLR3. **(B)** The mRNA levels of IFNs, including IFN-α, IFN-β, and IFN-γ. **(C)** The mRNA levels of pro-inflammatory cytokines, including IL-1β, IL-6, IL-8. Treatment with saline was used as the control. Data are expressed as means ± standard deviations (*n* = 5). Differences were evaluated with Two-tailed Student's *t*-test. ^#^*p* > 0.05, *p < 0.05, **p < 0.01, ***p < 0.001.

Among the PRRs expressed in the spleens (Figure 5A), DTMUV infection increased the mRNA levels of RIG-I, MDA5, and TLR3 significantly. And we further found that the levels of RIG-I and MDA5 were inhibited with Rapa treatment, whereas enhanced with 3-MA and CQ treatments in DTMUV-infected spleens, as compared to those with saline treatment. Besides, Rapa treatment increased the mRNA levels of TLR3, while 3-MA and CQ treatments decreased it compared to that in saline treatment. These results indicated that autophagy played a positive role in the expression of RIG-1 and MDA5, but a negative role in TLR3.

Among the IFNs expressed in the spleens (Figure 5B), DTMUV infection increased the mRNA levels of IFN-α (28.23-fold) and IFN-β (2.5-fold) significantly, in particular a bigger increase of the IFN-α level compared to that of IFN-β. Whereas, the level of IFN-γ was decreased in DTMUV-infected spleens. Furthermore, we found that the levels of IFN-α and IFN-β were inhibited with Rapa treatment, whereas enhanced with 3-MA and CQ treatments in DTMUV-infected spleens, as compared to those with saline treatment. But there were no significant effects on IFN-γ levels in the spleen tissue with autophagy regulator treatments.

Among the cytokines expressed in spleen tissues (Figure 5C), DTMUV infection only decreased the mRNA level of IL-8 significantly, whereas no effects on IL-1β and IL-6. We also found that Rapa treatment further inhibited the level of IL-8, while 3-MA and CQ treatments enhanced that compared to saline treatment, which indicated that the level of IL-8 was correlated to the autophagy responses. In addition, 3-MA and CQ treatments have no effects on the mRNA levels of IL-1β and IL-6, while Rapa treatment increased the levels of IL-1β significantly, but no effects on IL-6.

#### Expression of Immune Genes in the Brains

**Figure 6:**
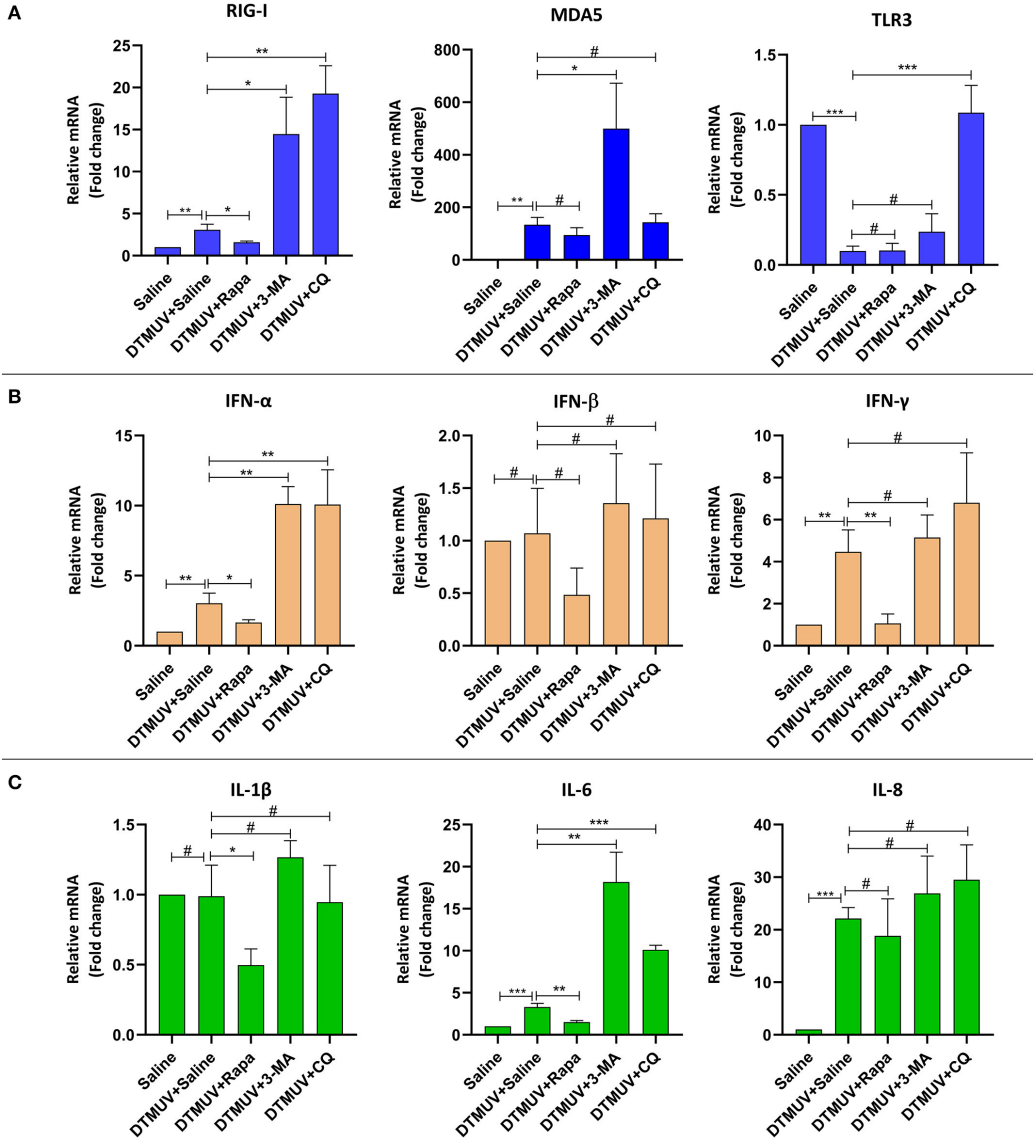
Expression of immune-related genes in the brains of ducks infected with DTMUV in the absence or presence of either Rapa, 3-MA, or CQ, respectively. Treatment with saline was used as the control. **(A)** The mRNA levels of PRRs, including RIG-I, MDA5, and TLR3. **(B)** The mRNA levels of IFNs, including IFN-α, IFN-β, and IFN-γ. **(C)** The mRNA levels of cytokines, including IL-1β, IL-6, IL-8. Data are expressed as means ± standard deviations (*n* = 5). Differences were evaluated with Two-tailed Student's *t*-test. ^#^*p* > 0.05, *p < 0.05, **p < 0.01, ***p < 0.001.

Among the PRRs expressed in brain tissues (Figure 6A), DTMUV infection increased the mRNA levels of RIG-I, MDA5, but decreased TLR3 significantly. And the level of RIG-I was inhibited with Rapa treatment, whereas enhanced with 3-MA and CQ treatments, as compared to saline treatment. In addition, Rapa treatment has no effects on the levels of MDA5 or TLR3. 3-MA treatment increased the levels of MDA5 but no effect on TLR3, whereas CQ treatments increased the levels of TLR3 but no effects on MDA5.

Among the IFNs expressed in brain tissues (Figure 6B), DTMUV infection increased the mRNA levels of IFN-α and IFN-γ significantly, but no effects on IFN-β. And the level of IFN-α was inhibited with Rapa treatment while enhanced with 3-MA and CQ treatments, as compared to saline treatment. But there were no significant effects on IFN-β levels with autophagy regulator treatments. In addition, Rapa treatments inhibited the level of IFN-γ, whereas 3-MA and CQ treatments have no effects on that.

Among the cytokines expressed in brain tissues (Figure 6C), DTMUV infection increased the mRNA levels of IL-6 and IL-8 significantly, but no effects on IL-1β. Furthermore, the level of IL-6 was inhibited with Rapa treatment, whereas enhanced with 3-MA and CQ treatments, as compared to saline treatment. In addition, Rapa treatment inhibited the level of IL-1β but no effects on IL-8. And 3-MA or CQ treatments have no effects on IL-1β and IL-8 levels.

## Discussion

Multiple evidence suggests that autophagy plays a crucial role in the life cycles of flaviviruses *in vitro* and *in vivo* (Ke, 2018). As we have investigated that autophagy promotes the replication of DTMUV *in vitro* (Hu et al., 2020), to further provide the clinical evidence on the effects of autophagy on DTMUV replication and pathogenesis, we utilized ducks as the animal model to study the role of autophagy in DTMUV-targeted organs.

We first found that DTMUV infection triggered autophagy in duck spleens and brains which were the target organs of DTMUV (Figure 1 and Figure S1). Autophagy always occurs in the target organs of virus infection. For instance, Dengue virus (DENV), another flavivirus, also has been reported to trigger autophagy in mice brains (Lee et al., 2013). Newcastle Disease Virus (NDV) and Avian Influenza A H5N1, RNA virus of other species, also trigged autophagy in their target organs, respectively (Sun et al., 2012, 2014). The trigged-autophagy in target organs might be caused by the amount of virus replication in these organs. Moreover, to study the effects of autophagy on DTMUV pathogenicity *in vivo*, we utilized autophagy regulators to adjust the host autophagic levels. Rapa has been investigated to be an effective enhancer of autophagy *in vivo*, such as mice (Lee et al., 2013) chickens (Sun et al., 2014). Our result in Figure 1 showed the protocol of Rapa treatment in this study successfully increased the autophagic level. 3-MA inhibits autophagy by blocking autophagosome formation via the inhibition of class III PI3K (Klionsky et al., 2016) and has been used to inhibit the autophagy induced by various Flaviviruses, such as Zika Virus (ZIKV) *in vitro* (Cao et al., 2017), DENV *in vitro* (Lee et al., 2008) and *in vivo* (Lee et al., 2013), classical swine fever virus (CSFV) *in vitro* (Pei et al., 2014). And our previous data has shown 3-MA treatment successfully inhibits DTMUV-induced autophagy *in vitro* (Hu et al., 2020). However, one case shows that prolonged treatment with 3-MA promotes autophagy under nutrient-rich conditions (Wu et al., 2010). But there is no evidence to show the promotion of 3-MA treatment in virus- or starvation-inducted autophagy. In this study, we treated ducks with 3-MA once every 12 h and for 72 h, which had a good efficiency for the inhibition of DTMUV-inducted autophagy in duck brains and spleens (Figure 1). And also, to eliminate the problem of the dual role of 3-MA in autophagy, we utilized another autophagy inhibitor, CQ, to block DTMUV-induced autophagy. CQ inhibits autophagy by decreasing autophagosome-lysosome fusion (Mauthe et al., 2018), and has been reported to block NDV-inducted autophagy in Chickens, which is a relative species to duck (Sun et al., 2014). In the current study, CQ enhanced the accumulation of LC3-II and p62 (Figure 1), indicting the successful inhibition of DTMUV-induced autophagy in ducks. Above all, the autophagy inducer and inhibitors adjusted autophagic levels in the two organs successfully, which meant that we could utilize these models to study the mechanism of the role of autophagy on DTMUV replication next.

DTMUV caused obvious gross pathologies and histopathologic lesions in duck spleens and brains (Figures 2, 3). These changes are consistent with other's reports (Lv et al., 2019; Sun et al., 2019a). And we first found that the levels of autophagy were positively correlated with the degree of tissue damages induced by DTMUV infection. The therapeutic effects of autophagy inhibitors also have been reported in ZIKV (Zhang et al., 2019a), DENV (Lee et al., 2013), NDV (Sun et al., 2014), and H5N1 (Sun et al., 2012) infection *in vivo*. Moreover, the tissue damage of spleens indicated that DTMUV infection might cause damage to the host immune system. Multiple evidence indicates that autophagy is a tool to regulate the innate immune system (Germic et al., 2019). So, autophagy inhibitors might alleviate spleen damage by adjusting the levels of innate immune responses. Like some other flaviviruses (Mustafá et al., 2019), DTMUV infection also counteracted the Blood-Brain Barrier and invaded the Central Nervous System (CNS). There is an opinion that the pathogenesis of CNS is always related to impaired autophagy (Nikoletopoulou et al., 2015). Our results also showed that brain damage was related to the levels of autophagy. We provided some references for the therapeutic developments of flaviviruses-caused neurologic symptoms.

The levels of tissue damage are always related to virus replication in the corresponding tissues. In the current study, we found that autophagy regulator treatments affected DTMUV replication significantly in the target tissues. The formation of autophagosomes has been reported to be required for the maturation of infectious dengue virus production (Mateo et al., 2013). And also, autophagosomes supply places for the formation of membrane structures of hepatitis C virus (HCV) (Mohl et al., 2016). Besides, some flaviviruses, including HCV (Ren et al., 2016; Shrivastava et al., 2016), DENV (McLean et al., 2011), and Japanese encephalitis virus (Tasaki et al., 2016), use autophagic membranes for their release via the exosomal pathway through multivesicular bodies (Shrivastava et al., 2011; Metz et al., 2015), and these may be related to the process of the fusion between autophagosomes and lysosomes. Therefore, in this study, Rapa and 3-MA might affect the replication of DTMUV in target tissues by regulating the formation of autophagosomes. 3-MA also has been reported to reduce DENV replication in mice by autophagy pathway (Lee et al., 2013). CQ is an autophagy inhibitor by inhibiting the autophagosome-lysosome fusion and might reduce DTMUV replication by targeting this pathway. Similar results are also found in ZIKV-infected mice with CQ treatment (Zhang et al., 2019a). Moreover, our lab's previous data invested that p62 regulated the innate antiviral response in DTMUV-infected cells (Hu et al., 2020). Hence, CQ treatments might also inhibit DTMUV replication by causing the accumulation of p62, and then affecting the host antiviral response *in vivo*. In addition, CQ also has some antiviral activities by autophagy-independent pathways. CQ is weak base, and meddles in protein processing, and degradation by alkalifying the acidic organelles like Golgi vesicles, endosomes, lysosomes (Gratton et al., 2019). And the low pH is essential for the entry, the viral RNA release, and the exit of flaviviruses, like ZIKV, DENV, HCV, and others (Tscherne et al., 2006; Sánchez-San Martín et al., 2009; Zheng et al., 2014; Persaud et al., 2018). DTMUV also has been reported to enters BHK-21 cells by a low pH-dependent endosomal pathway (Baloch et al., 2019). Therefore, CQ treatment might inhibit DTMUV replication by blocking the pH-dependent stages of DTMUV replication. Further study needs to be done for the mechanism of the therapeutic effects on Flavivirus infection.

How did autophagy affect DTMUV replication in spleens or brains? Autophagy has been seen to mediate the innate immunity through the secretion of interferon and inflammation (Deretic et al., 2013). So, we tested the expression levels of innate immune genes in the two tissues with autophagy regulator treatments. There is a limitation of utilizing pharmaceutical autophagy regulators in the research on the interface between autophagy and the immune system. Because chemical inhibitors or inducers always affect multiple cellular pathways, and some chemical regulators can affect immune responses in an autophagic-independent manner (Klionsky et al., 2016). For instance, PI3K is not only the target of many autophagy inhibitors like 3-MA, Wortmannin and LY294002, but also the regulator of Toll-like receptor (TLR)-mediated inflammatory responses (Kuo et al., 2006; Guiducci et al., 2008). 3-MA has been reported to regulate inflammatory response by PI3K-Akt-Glycogen pathway rather than autophagy (Lin et al., 2012). Another type of autophagy inhibitors, including CQ and Bafilomycin A1, are targeting endosomal acidification which is related to the signaling of endosomal TLRs (Rutz et al., 2004; Hart et al., 2005). Actually, genetic approaches and methods based on the specific depletion of ATG proteins from different autophagy functional clusters are the best experimental strategy for this experiment (Echavarria-Consuegra et al., 2019). But it is hard to perform RNA silencing or other protein- or gene-specific targeting technologies in animals of the duck species which is an unconventional animal model. Therefore, to eliminate these problems, we set up both autophagy-enhanced and autophagy-inhibited treatments and then screened out the immune genes whose expression levels varied with autophagy levels. We found that the mRNA levels of RIG-I, MDA5, TLR3, IFN-α, IFN-β, and IL-8, were changed with autophagy levels altered by autophagy regulators in the spleen, whereas the mRNA levels of RIG-I, IFN-α, and IL-6 in the brain. The expression of type I IFNs is controlled by upstream PRR signaling pathways, including the retinoic RIG-I-like receptor (RLR) family and the TLR family (Tian et al., 2019). In the current study, we found that autophagy played a negative role in the expression levels of RLPs and type I IFNs in both duck spleens and brains. HCV-mediated and DENV-mediated autophagy also have been reported to suppress RIG-I signaling and type I IFN production (Ke and Chen, 2011; Shrivastava et al., 2011). But the mechanism of autophagy -suppressed RLR pathways and type I IFNs and the different responses in different organs are still unknown. There was an interesting finding that TLR3 levels were negatively correlated with type I IFN levels in the spleens. The signaling of endosomal TLRs might be affected by autophagy-associated membrane fusion events. However, the interaction between autophagy and endosomal TLRs is still unclear and controversial. Autophagy has been reported to be a positive role in TLR7-mediated and TLR9-mediated type I IFN production (Zhou et al., 2012; Hayashi et al., 2018), whereas a negative role in TLR7-mediated type I IFNs in Enterovirus 71 (EV71) and coxsackievirus A16 (CA16) infections (Song et al., 2018). The role of autophagy on TLR3-mediated type I IFNs in DTMUV infection needs further study in the future. Cytokines play protective or destructive roles in response to virus infection, and autophagy plays critical roles in virus-mediated inflammation secretion (Zhong et al., 2016; Qian et al., 2017). In the current study, we found autophagy inhibitors enhanced IL-8 mRNA level in the spleen, whereas enhanced IL-6 levels in the brain. Some other reports shows that autophagy inhibition reduces the production of IL-8 (Harris et al., 2011; Luo et al., 2015). We thought this difference might because of the different host species, different pathogens, or different organs. IL-6 is not only a factor involved in the immune response but also plays many critical roles in the nervous system (Erta et al., 2012). And IL-6 has been reported to be associated with the West Nile virus (WNV)-caused neuroinflammatory (Kumar et al., 2010). The changes of IL-6 level special in the brain might be related to DTMUV-caused neurologic symptoms (Figure 3).

**Figure 7:**
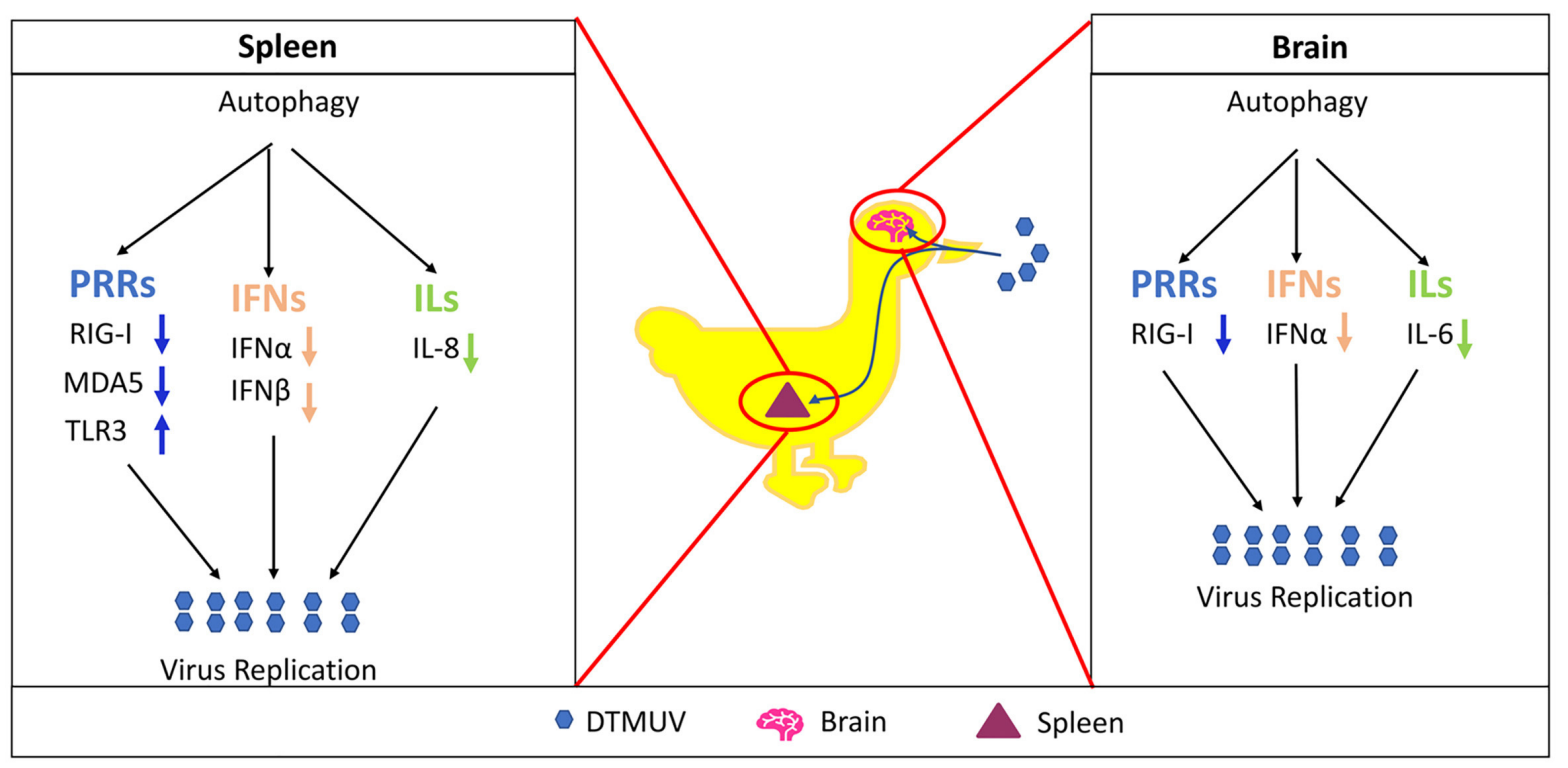
Proposed model of autophagy promotes DTMUV replication and tissue damages *in vivo*.

In summary, as shown in Figure 7, the current study demonstrated that DTMUV-trigged autophagy facilitated DTMUV replication, aggravated the developments of pathological symptoms and possibly counteracts the host's innate immunity response *in vivo*. However, the mechanism of the different autophagy-mediated immune responses in different tissues is still unknown. Above all, our study supports further clinical evidence to use autophagy-related therapies against DTMUV infection and gives some references for the developments of the treatments for other flavivirus infections.

## Data Availability Statement

All datasets generated for this study are included in the article/Supplementary Material.

## Ethics Statement

The animal study was reviewed and approved by the committee of experiment operational guidelines and animal welfare of Sichuan Agricultural University, China (the approved permit number is XF2014-18).

## Author Contributions

ZH and RJ conceived and designed the experiments. ZH and YP guided and conducted the experiment and analyzed the data. ZH wrote the original draft preparation. RJ and YP reviewed and edited the manuscript. AC, MW, SC, DZ, ML, QY, YW, XZhao, SZ, ZY, YY, LZ, and YL contributed materials. XZhang, JH, SM, XO, BT, LP, and MR helped to analyze the data. All authors read and approved the final manuscript.

### Conflict of Interest

The authors declare that the research was conducted in the absence of any commercial or financial relationships that could be construed as a potential conflict of interest.
