# Corrigendum: Autophagy Is a Potential Therapeutic Target Against Duck Tembusu Virus Infection *in Vivo*

**DOI:** 10.3389/fcimb.2021.641825

**Published:** 2021-02-19

**Authors:** Zhiqiang Hu, Yuhong Pan, Anchun Cheng, Xingcui Zhang, Mingshu Wang, Shun Chen, Dekang Zhu, Mafeng Liu, Qiao Yang, Ying Wu, Xinxin Zhao, Juan Huang, Shaqiu Zhang, Sai Mao, Xumin Ou, Yanling Yu, Ling Zhang, Yunya Liu, Bin Tian, Leichang Pan, Mujeeb Ur Rehman, Zhongqiong Yin, Renyong Jia

**Affiliations:** ^1^ Institute of Preventive Veterinary Medicine, Sichuan Agricultural University, Wenjiang, China; ^2^ Avian Disease Research Center, College of Veterinary Medicine of Sichuan Agricultural University, Wenjiang, China; ^3^ Key Laboratory of Animal Disease and Human Health of Sichuan Province, Sichuan Agricultural University, Wenjiang, China

**Keywords:** DTMUV, autophagy, spleen, brain, tissue damage, replication, immune response

In the original article, there was a mistake in **Figure 3** as published. This figure contains the wrong slides of microscopy. The corrected **Figure 3** appears below.

The authors apologize for this error and state that this does not change the scientific conclusions in any way. The original article has been updated.

**Figure 3:**
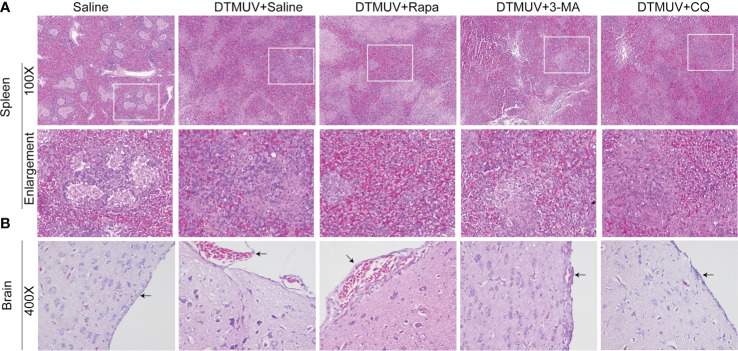
Hematoxylin and eosin staining of cells in the spleens **(A)** and the brains **(B)** of ducks infected with DTMUV in the absence or presence of either Rapa, 3-MA, or CQ, respectively. Treatment with saline was used as the control. Images shown were representative from five ducks in each group. The images were with 100X magnification for spleens and 400X for brains. Black arrows: blood cells in the meninx.

