# Supplementary material for: Autophagy Is a Potential Therapeutic Target Against Duck Tembusu Virus Infection in vivo
Source: Front Cell Infect Microbiol. 2020 Apr 15;10:155. doi: 10.3389/fcimb.2020.00155 (PMC7174708; doi:10.3389/fcimb.2020.00155)
Supplement: Supplementary file 1 [file Data_Sheet_1.docx]

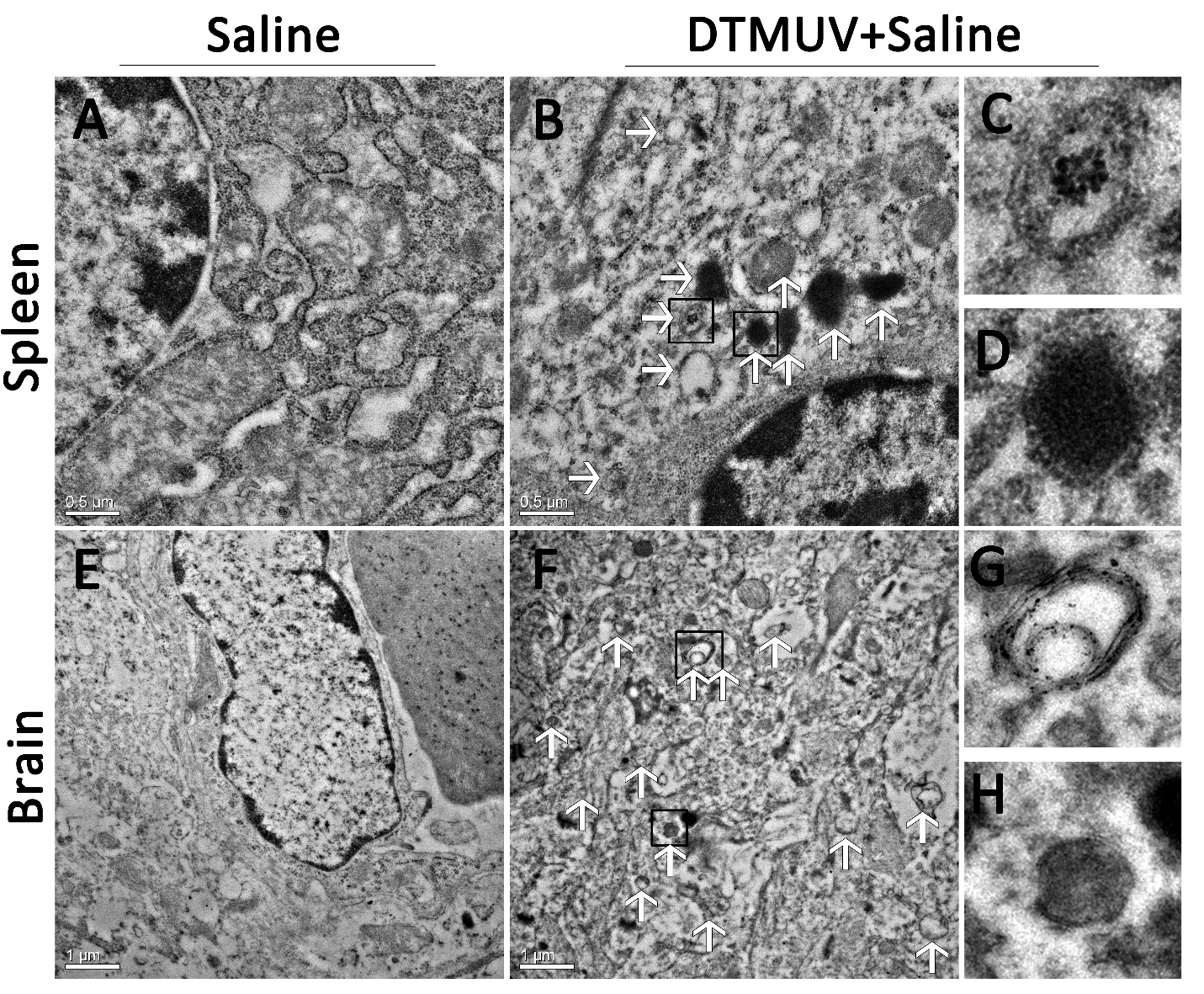


Figure S1 Observation of autophagy-like vesicle formation by TEM in the duck spleens (A-E) and brains (F-H). (A, E) Spleen and brain respectively, no obvious autophagy-like vesicles observed in the only saline-treated group. (B, F) Spleen and brain respectively, obvious autophagosome-like and autolysosome-like vesicles in the DTMUV-infected group. (C, D) A higher-magnification view of autophagosome-like vesicle and autolysosome-like vesicle from (B) respectively. (G, H) A higher-magnification view of autophagosome-like vesicle and autolysosome-like vesicle from (F) respectively. White arrow: autophagosome-like vesicles and autolysosome-like vesicles. Images shown were representative from five ducks in each group.
